# Development and Evaluation of 3D-Printed Losartan Potassium Tablets Using Semi-Solid Extrusion: The Effect of Geometry, Drug Loading and Superdisintegrant

**DOI:** 10.3390/ph18101504

**Published:** 2025-10-07

**Authors:** Aleksandra Vojinović, Đorđe Medarević, Gordana Stanojević, Dušica Mirković, Snežana Mugoša, Ivana Adamov, Svetlana Ibrić

**Affiliations:** 1 Institute for Medicines and Medical Devices of Montenegro, 81000 Podgorica, Montenegro; gordana.boljevicstanojevic@cinmed.me (G.S.); snezana.mugosa@cinmed.me (S.M.); 2 Faculty of Pharmacy, University of Belgrade, 11000 Belgrade, Serbia; djordje.medarevic@pharmacy.bg.ac.rs (Đ.M.); ivana.adamov@pharmacy.bg.ac.rs (I.A.); svetlana.ibric@pharmacy.bg.ac.rs (S.I.); 3 Medical Faculty, Military Medical Academy, 11000 Belgrade, Serbia; dusicamirkovc11@gmail.com

**Keywords:** semi-solid extrusion, 3D printing, losartan potassium, printability, personalized medicine, oral solid dosage form, polymer

## Abstract

**Background/Objectives:** Semi-solid extrusion (SSE) three-dimensional (3D) printing offers a versatile approach for fabricating personalized oral dosage forms. This study aimed to develop and optimize losartan potassium tablets produced via SSE 3D printing, focusing on the effects of polymer composition, tablet geometry, drug loading, and superdisintegrant concentration on printability and performance characteristics. **Methods:** Formulations containing hydroxypropyl methylcellulose (HPMC) 4500 at various concentrations were evaluated for suitability in an ethanol–water (9:1 *v*/*v*) solvent system. The optimized formulation (5% *w*/*w* HPMC 4500) was used to print tablets with varying shapes, drug loadings (5–15% *w*/*w*; approximately 50–150 mg losartan potassium per tablet), and croscarmellose sodium concentrations (0–3% *w*/*w*). Printed tablets were characterized for dimensional accuracy, mass uniformity, disintegration time, and drug release behavior. Drug release kinetics were modeled to elucidate the release mechanism. **Results:** All SSE-printed tablets exhibited excellent dimensional precision (SD < 0.8 mm) and mass uniformity (SD < 0.12 g). Increasing drug loading enhanced the initial release rate, reaching up to 63% in 45 min for 15% loading. The addition of 1% croscarmellose sodium reduced disintegration time to approximately 25 min. Drug release profiles were best described by the Korsmeyer–Peppas model (R^2^ > 0.96), indicating diffusion-controlled release. **Conclusions:** SSE 3D printing demonstrated robustness and flexibility in producing losartan potassium tablets with consistent quality, tunable release properties, and strong potential for personalized pharmaceutical manufacturing.

## 1. Introduction

Traditional pharmaceutical manufacturing primarily relies on batch production and follows a standardized “one-dose-fits-all” model. Although this approach supports large-scale efficiency, it lacks the adaptability required to address the growing need for individualized treatments. Emerging knowledge from fields such as pharmacogenomics, pharmacometrics, and clinical pharmacology has highlighted significant differences in how patients respond to therapies—variations influenced by genetic polymorphisms, age, sex, body weight, comorbidities, and other individual factors. These findings have driven a shift toward personalized medicine, in which drug formulations are customized to meet each patient’s specific needs in terms of dosage, release characteristics, and mode of administration [1,2,3].

One of the most promising technologies enabling this shift is three-dimensional (3D) printing, which has gained increasing attention in pharmaceutical sciences for its potential to fabricate customized dosage forms with precise control over structure and composition. Since the FDA approval of Spritam^®^, the first 3D-printed drug product, there has been a surge in research and development efforts exploring various 3D printing techniques—including fused deposition modeling (FDM), binder jetting, vat polymerization, and semi-solid extrusion (SSE)—for drug delivery applications [4,5,6].

Semi-solid extrusion (SSE), in particular, has become a promising technique for producing oral drug delivery systems, largely due to its compatibility with a wide variety of excipients and its ability to operate at low temperatures, preserving the stability of thermolabile drug substances [6,7,8]. In contrast to fused deposition modeling (FDM), which requires high processing temperatures and specialized polymer grades, SSE enables extrusion of gel- or paste-based formulations using conventional pharmaceutical polymers, such as hydroxypropyl methylcellulose (HPMC), polyethylene glycols (PEGs), gelatin, etc. [8,9,10].

The versatility of SSE allows the formulation of immediate-release tablets [11,12], extended-release matrices [13], polypills [14], chewable dosage forms [15,16], and drug products designed for narrow therapeutic windows or pediatric use [17,18]. Moreover, this technology supports dose flexibility, geometry customization, and decentralized manufacturing, aligning with the broader goals of industry and on-demand production in hospital or pharmacy settings [19,20].

Despite its potential, successful adoption of SSE in pharmaceutical manufacturing depends heavily on selecting the right combination of excipients and polymers, control over formulation rheology, and optimization of printing parameters [21,22,23]. Printability remains one of the most critical attributes in SSE, influencing not only process feasibility but also product uniformity, drug release behavior, and mechanical characteristics [24,25]. Research indicates that aspects such as polymer concentration, solvent system, and inclusion of viscosity enhancers or disintegrants can significantly impact the extrusion process, shape maintenance, and interlayer cohesion [26,27].

Losartan potassium is an angiotensin II receptor antagonist (ARB) that selectively blocks the AT_1_ receptor subtype, thereby reducing vasoconstriction, aldosterone secretion, and blood pressure. It is widely prescribed for the treatment of hypertension, heart failure, and for renal protection in patients with type 2 diabetes and proteinuria. Losartan potassium has an oral bioavailability of approximately 33%, with peak plasma concentrations reached within 1–2 h and a terminal elimination half-life of about 2 h for the parent drug and 6–9 h for its active metabolite, EXP3174. Standard clinical doses range from 25 to 100 mg daily, either as a single dose or divided doses, depending on patient response. The drug is freely soluble in water, stable under the mild temperature conditions used in SSE, and is suitable for flexible dose adjustment, making it an appropriate candidate for 3D printing of personalized oral dosage forms. Losartan potassium was selected as a model drug due to its clinical relevance as an angiotensin II receptor antagonist widely prescribed for hypertension management, its moderate aqueous solubility (BCS Class II), and its sensitivity to formulation and manufacturing parameters that may influence dissolution performance. These properties make it suitable for evaluating the influence of geometry, drug loading, and excipient selection on release kinetics in 3D-printed dosage forms.

While earlier research explored the effects of drug loading or geometry individually, it remains essential to conduct an integrated investigation that combines formulation screening, geometric variability, drug dose adjustment, and disintegration kinetics, particularly using conventional pharmaceutical-grade polymers suitable for regulatory translation.

Therefore, the aim of this study was to develop and evaluate 3D-printed losartan potassium tablets using the SSE technique. The research focused on the following:

- Identifying suitable polymer–solvent systems for printability;
- To evaluate the physicochemical properties of losartan potassium in the context of formulation design;
- Investigating the effects of tablet geometry, drug loading, and superdisintegrant concentration on drug release and disintegration, and;
- Applying kinetic modeling and spectroscopic analysis to understand release mechanisms and component compatibility;
- Develop improved or alternative formulations compared to existing commercial products considering quantitative and qualitative drug attributes.

This study represents the first systematic combination of geometry, drug loading, and superdisintegrant variation in HPMC-based SSE printing for losartan potassium, integrating both dissolution modeling and printability evaluation.

## 2. Results and Discussion

### 2.1. Printability Assessment of the Polymer–Solvent–Excipient Mixtures

Critical considerations in the printing process:

- Infill Density and Pattern: A 100% infill density and linear pattern were chosen to ensure mechanical integrity and prevent internal collapse, particularly during solvent loss. These settings also support reproducible drug release by minimizing porosity variation.
- Printing and Travel Speeds: A print speed of 15 mm/s was optimal for maintaining uniform deposition without filament discontinuity. Reduced initial layer speed (11 mm/s) was essential for proper anchoring of the base layer to the build plate.
- Cooling and Layer Adhesion: The fan was set to maximum (100%) to accelerate surface drying between layers, promoting strong interlayer bonding and dimensional stability.

The printability of each polymer–solvent–excipient mixture was evaluated through qualitative observations during and after extrusion. Key parameters included the homogeneity of the mixture, flow behavior, the presence and persistence of air bubbles, extrusion smoothness, and the dimensional stability of printed constructs. Formulations that exhibited excessive spreading, interlayer detachment, or nozzle clogging were deemed unsuitable for semi-solid extrusion (SSE). Furthermore, formulations that produced irregular lines or collapsed structures during layer deposition were identified as having insufficient plasticity or cohesion.

The results, summarized in Table 1, demonstrated clear differences in behavior depending on polymer type, concentration, and presence of excipients.

**Figure 1:**
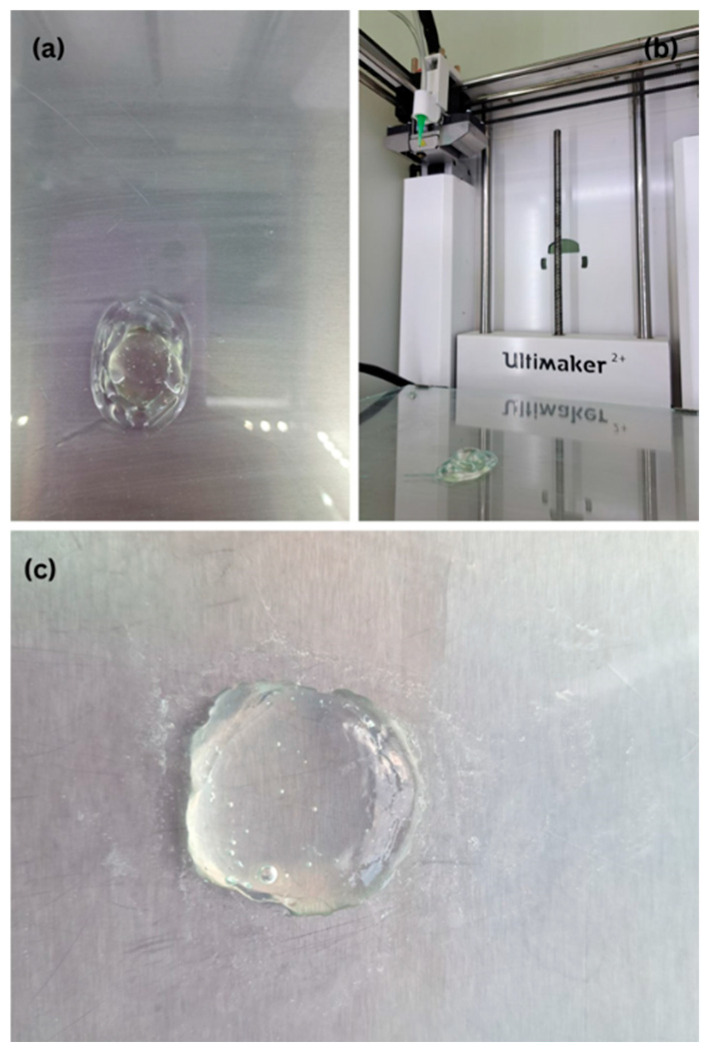
3D printed tablets using Benecel™ K35M (2%) + Croscarmellose sodium/Water (4%): (**a**) rectangle shape, (**b**) rectangle shape side view on building plate, (**c**) round tablet shape.

Formulations based on Benecel™ K35M, a high-viscosity grade of HPMC, exhibited suboptimal printability characteristics. At lower concentrations (1% *w*/*w*), the mixtures were overly flowable, lacking the viscosity required for defined structural deposition during printing. At higher concentrations (2% *w*/*w*), acceptable viscosity was achieved; however, this was accompanied by a high presence of entrapped air bubbles, likely due to the high molecular weight and hydration dynamics of the polymer. Even at increased croscarmellose sodium levels (4%), which did reduce bubble formation and increase viscosity, the mixtures exhibited significant shape spreading and poor plasticity (Figure 1). These properties prevented precise layer-by-layer deposition and led to poor structural integrity post-printing. Consequently, Benecel™ K35M was excluded from further formulation development.

Formulations containing HPMC 615, another high-viscosity cellulose ether, revealed similar limitations. Despite its adequate thickening behavior, aqueous mixtures (10–12%) with or without croscarmellose sodium suffered from significant bubble entrapment and poor interlayer adhesion, likely due to its inherent gelation and surface properties. When ethanol-water mixtures (50:50 *v*/*v*) were used to improve solubility and reduce bubble content, inhomogeneous systems were obtained, with visible phase separation and poor dispersion of excipients, particularly in the presence of croscarmellose sodium. These mixing difficulties, combined with rheological instability, rendered HPMC 615 unsuitable for the printing process.

On the other hand, HPMC 4500 emerged as the most suitable polymer for SSE-based 3D printing. A 5% (*w*/*w*) solution of HPMC 4500 in an ethanol–water mixture (9:1 *v*/*v*) with 1% croscarmellose sodium demonstrated favorable flowability, manageable viscosity, and adequate structural retention. Slight initial mixing difficulties were observed, attributed to the semi-polar nature of the solvent system and partial ethanol evaporation; however, these were resolved with controlled agitation and short settling time. The formulation maintained its structure upon extrusion, supporting successful construction of printed units with consistent morphology.

HPMC 4500 was selected as the polymer for the semi-solid formulation, commonly used in conventional pharmaceutical production of orally administered drugs as a stabilizer for suspensions and emulsions, a viscosity-increasing agent, a binder, and an excipient in coating and film formulations. HPMC 4500 is soluble in water and ethanol. A review of studies related to 3D printing via semi-solid extrusion revealed that HPMC is the most commonly used polymer for semi-solid formulations. During testing of formulations with different compositions, HPMC concentration of 5% proved to be optimal for achieving the best viscosity and extrusion performance. A mixture of ethanol and water (9:1 *V*/*V*) was used as the solvent for the active substance and excipients. During the testing of various formulations (with purified water and ethanol-water mixtures in different volume ratios), this mixture proved to be the most effective for dissolving HPMC 4500, and the formulation using this solvent showed the best extrusion properties. Additionally, scientific studies support this choice [12].

In addition to polymer selection, solvent evaporation during printing proved to be a notable factor. Ethanol’s boiling point (78 °C) was surpassed by the operational temperature of the extrusion nozzle, potentially altering viscosity during the process and emphasizing the need for optimized temperature control and solvent composition.

PEG 6000 serves as a plasticizer in the formulation, as it provides viscoelastic properties that enable uniform extrusion of the paste through the nozzle during printing. A concentration of 15% was used [28].

SiO_2_ was added as a viscosity-enhancing agent, with the aim of improving the appearance and fidelity of the printed form. Scientific studies have reported formulations with SiO_2_ content ranging from 0.5% to 2% [29]. When printing formulations containing SiO_2_, improved extrusion, more precise printing, and better structural integrity of the printed shape were observed compared to formulations without this excipient. A concentration of 1% was used.

Optimal printability was observed in formulations that maintained continuous flow through the nozzle without pulsation, retained shape after deposition, and allowed for reproducible layer stacking. These attributes were found predominantly in systems based on HPMC 4500 with ethanol–water solvent and low-to-moderate levels of croscarmellose sodium. Overall, printability assessment served as a critical filter for formulation selection prior to mechanical and functional evaluation (Table 2).

### 2.2. Uniformity of Mass and Dimension of Printed Tablets

The dimensional analysis of printed tablets confirmed that the obtained values closely matched the predefined parameters set in the slicing software. Across all geometries and formulations, the measured diameter and thickness values were in strong agreement with the intended design specifications, demonstrating high precision and reproducibility of the semi-solid extrusion (SSE) 3D printing process (Table 3).

It is worth emphasizing that changes in the concentration of losartan potassium had minimal impact on flow properties, viscosity, or printing outcome, suggesting that the polymer matrix dominates the rheological behavior in these systems. Similar conclusions have been drawn in earlier research, which highlight the dominant influence of polymer concentration and network density on the printability and mechanical performance of SSE formulations [30,31].

This dimensional consistency observed in the printed tablets highlights not only the precision of the printing hardware and software coordination but also the effectiveness of optimizing formulation viscosity and extrusion parameters. The tablets displayed a gelatinous yet structurally coherent texture, characteristic of hydrogel-based systems post-printing, suitable for handling and subsequent testing.

**Figure 2:**
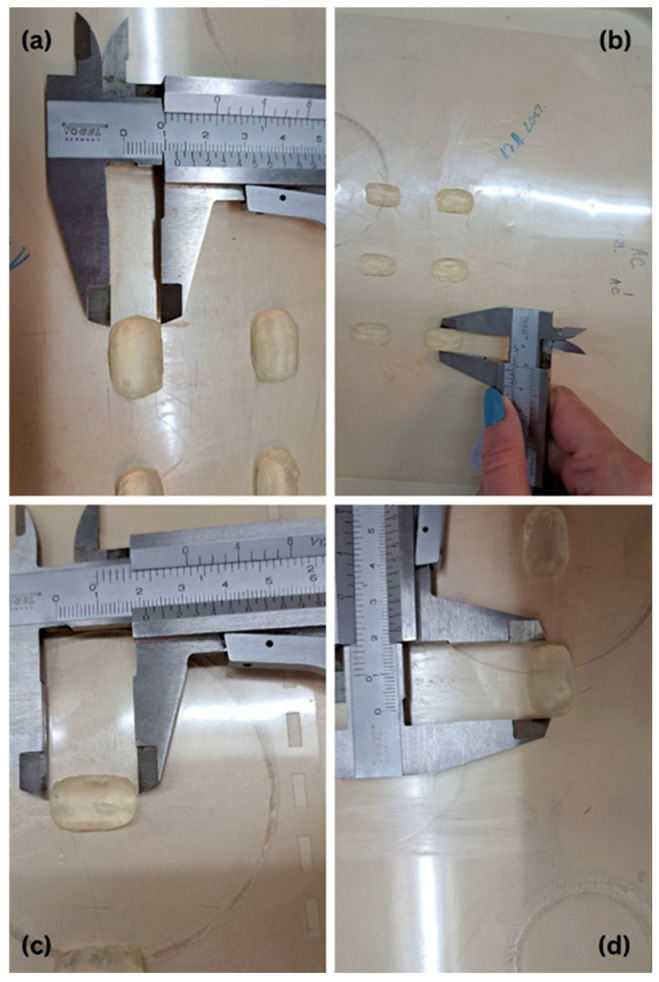
Three-dimensional-printed rectangle-shaped tablets F1 measured using a digital caliper: (**a**,**c**) width, (**b**,**d**) length.

**Figure 3:**
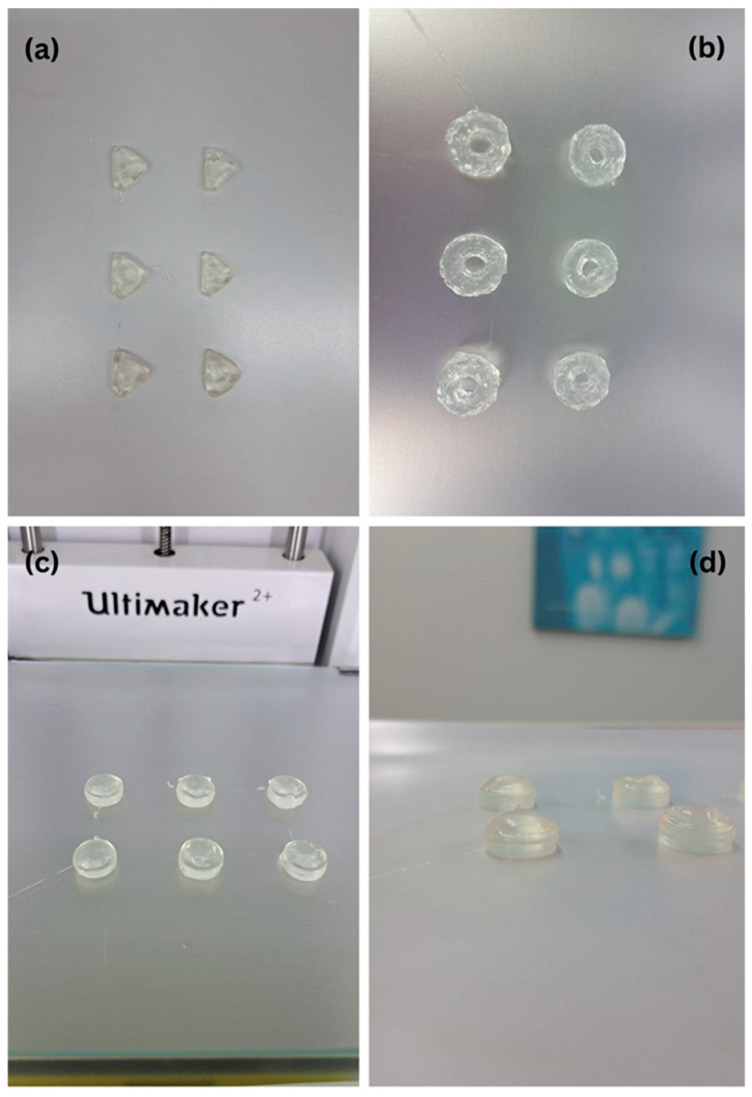
Three-dimensional-printed tablets (various shapes): (**a**) triangle shape, (**b**) doughnut shape, (**c**,**d**) round shape.

**Figure 4:**
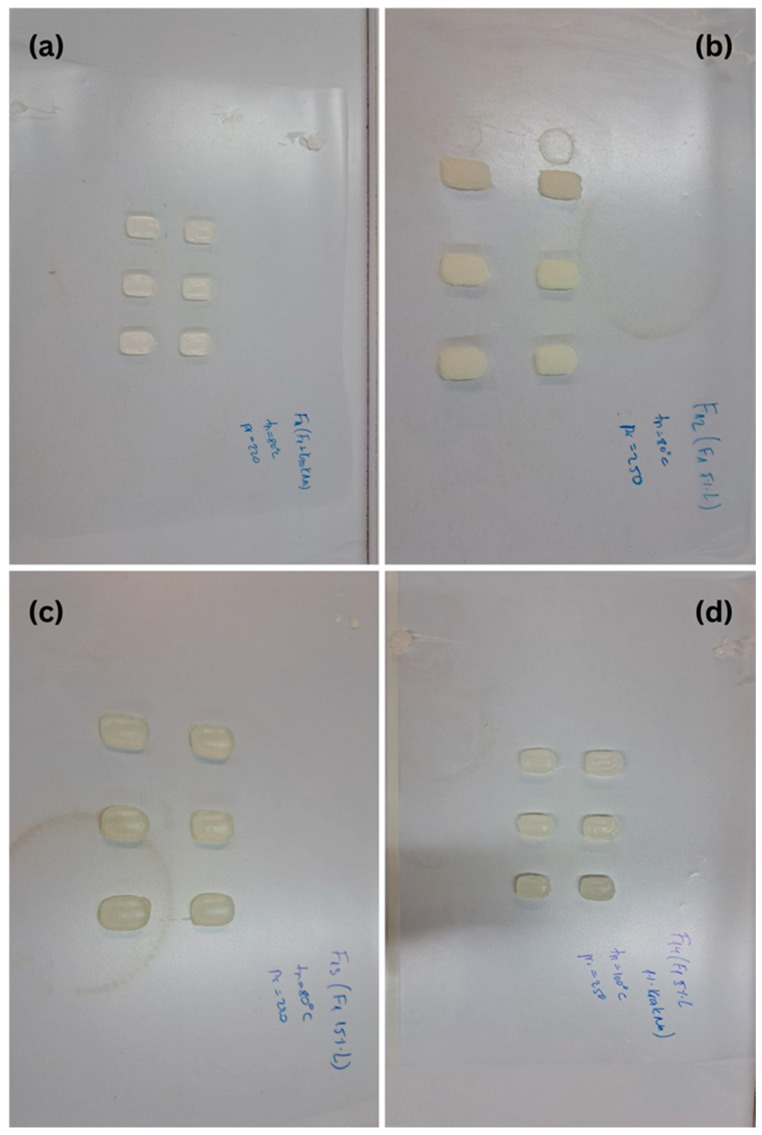
Three-dimensional-printed tablets rectangle shape (formulations F11–F14): (**a**) F11, (**b**) F12, (**c**) F13, (**d**) F14.

Representative images of the printed tablets are shown in Figure 2, Figure 3 and Figure 4, illustrating the reproducibility of geometry and surface definition across various shapes and formulations. These visual results further support the structural integrity and dimensional control achieved through the applied SSE printing protocol.

The average mass and standard deviation (SD) values for all printed formulations are presented in Table 4. The data demonstrate generally consistent mass production within individual formulations, as shown by relatively low standard deviations. However, notable differences in average tablet mass were observed between formulations, likely due to variations in formulation composition and tablet geometry.

Formulations F1–F4, which share the same composition but differ in design, showed mass values ranging from 0.398 g (F2, triangle) to 0.513 g (F4, circle), in line with their respective volumetric designs. Among them, formulation F1, containing 10% losartan potassium in a rounded rectangular shape, exhibited the most consistent mass uniformity, with the lowest standard deviation (0.026 g), indicating excellent reproducibility of both formulation handling and deposition accuracy.

Formulations F11–F14, which included croscarmellose sodium and variations in active substance content (5%, 10%, and 15%), had overall higher masses, with F13 reaching 0.821 g. Increased solid content and reduced solvent proportion contributed to these elevated values. Larger standard deviations, particularly in F12 (0.107 g) and F13 (0.116 g), may be explained by the higher viscosity of the mixtures and greater extrusion resistance, which can introduce slight variations during deposition.

Croscarmellose sodium is usually used in tablet formulation in concentrations 0.5–5.0% [32] and has shown binder/filler properties to maintain the shape after extrusion and enhance printability in semisolid extrusion [33].

Overall, the results demonstrate that tablet mass was well-controlled across all formulations, confirming the precision and reliability of the SSE 3D printing process for individualized drug delivery systems.

### 2.3. Drug Release

#### 2.3.1. Influence of Tablet Shape on Drug Release

The drug release profiles of losartan potassium from different tablet geometries (rounded rectangle, triangle, doughnut, and circle) were analyzed to assess the impact of shape on dissolution behavior and presented in Figure 1. Although the formulation composition remained constant (F1–F4), the surface area and volume varied depending on the geometry, potentially influencing drug release dynamics.

It has been established that inconsistencies in the dimensions and shape of printed tablets or other dosage forms can lead to inaccuracies in drug dosage resulting in overdosing or underdosing [34]. Also, variations in shape can affect active substance dissoluton profiles and therefore bioavailability [35,36].

**Figure 5:**
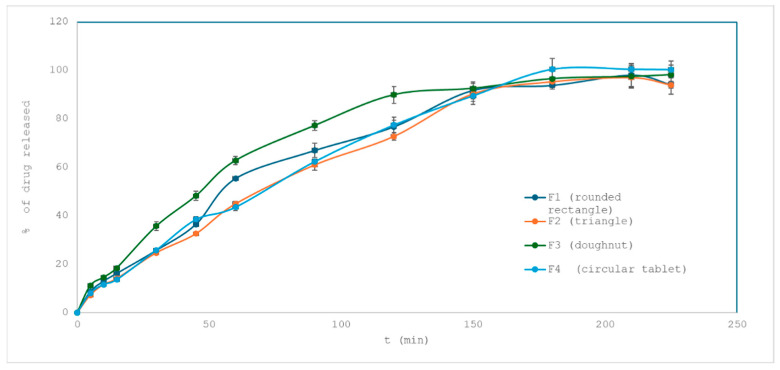
Drug release from formulation F1–F4 (influence of tablet shape).

To put this in context, previous studies have indeed demonstrated that geometry can markedly affect drug release. Goyanes and co-workers printed five different shapes (cube, sphere, cylinder, pyramid, and torus) of PVA-based tablets and found that drug release correlated strongly with the surface area-to-volume ratio of the shape. Notably, the torus (doughnut-like) and other high surface-area forms released drug more rapidly, and the authors concluded that geometry can be used as a design parameter to tune release kinetics [37]. Another example is the work of Yu and colleagues, who fabricated multi-layer doughnut-shaped tablets by a binder-jetting 3D printer; this design achieved a near zero-order release profile, leveraging the geometric form to control the surface area exposure over time [38] (Figure 5).

Comparison of dissolution profiles was performed using the similarity factor (f_2_). Values above 50 indicate comparable release kinetics. The f_2_ values calculated between the round tablet (reference) and the other shapes were 66.67 (rounded rectangle), 73.14 (triangle), and 52.50 (doughnut), suggesting that triangle and rounded rectangle tablets exhibit similar dissolution behavior to the round standard, while the doughnut showed moderate deviation, likely due to its greater surface area. Nevertheless, calculated f_2_ values suggest similar dissolution behavior throughout all printed shapes, in relation to desired properties.

Although overall differences between geometries were modest, the doughnut design showed a lower f_2_ similarity value (~52.5) compared to other shapes. This finding suggests that the internal void space provided an additional surface for water penetration and enabled hydration from both external and internal fronts. The dual-front swelling likely accelerated polymer erosion and drug release in the initial phases, illustrating that internal cavities or porous architectures can modulate dissolution kinetics even within diffusion-controlled hydrophilic matrices. Therefore, while geometry exerted only a secondary effect compared to formulation parameters, designs with internal voids (e.g., doughnut-like shapes) highlight the potential of 3D printing to fine-tune release behavior through structural features.

These differences in dissolution may be partly attributed to geometric characteristics. As shown in Table 2, the surface-area-to-volume ratios for triangle, rounded rectangle, and doughnut tablets were higher (0.786, 0.778, and 0.800 mm^−1^, respectively) compared to the round tablet (0.667 mm^−1^). Greater surface exposure may accelerate water penetration and matrix hydration, resulting in faster drug release.

To better understand the underlying mechanisms of drug release, dissolution profiles were fitted to standard mathematical models (Table 5).

The best fit was observed with the Korsmeyer–Peppas model (R^2^ > 0.96 for all geometries), indicating a diffusion-driven mechanism, potentially combined with matrix erosion. The Higuchi model also provided a strong fit (R^2^ ~ 0.96), supporting diffusion as the predominant release pathway. Zero-order and first-order models showed slightly lower determination coefficients (R^2^ = 0.73–0.95), suggesting non-constant release rates and complex kinetics. The Hixson–Crowell model, which accounts for changing surface area due to tablet erosion, had the lowest R^2^ values across most shapes.

The shape of the tablet affects both the rate and mechanism of drug release, even when the formulation remains constant. These findings highlight the importance of geometric optimization in 3D-printed drug delivery systems and demonstrate the utility of mathematical modeling for mechanistic interpretation.

In sustained release matrix tablets of 100 mg losartan potassium HCl fabricated by direct compression method, formulations with HPMC K100 M and Affinisol^®^ extended the drug release up to 12 h [39].

Research into the extended-release properties of losartan sodium indicated that utilizing a balanced ratio of hydrophobic and hydrophilic polymers can achieve controlled drug release for up to 12 h [40].

#### 2.3.2. Effect of Superdisintegrant on Drug Release

To evaluate the impact of superdisintegrant addition on the drug release rate of losartan potassium from 3D-printed tablets, dissolution profiles of formulations differing solely in the presence and concentration of croscarmellose sodium were compared. Formulations F1 and F11 shared the same composition (10% drug loading), shape (rounded rectangle), and printing conditions, with F11 containing 1% croscarmellose sodium and F1 being free of any superdisintegrant.

**Figure 6:**
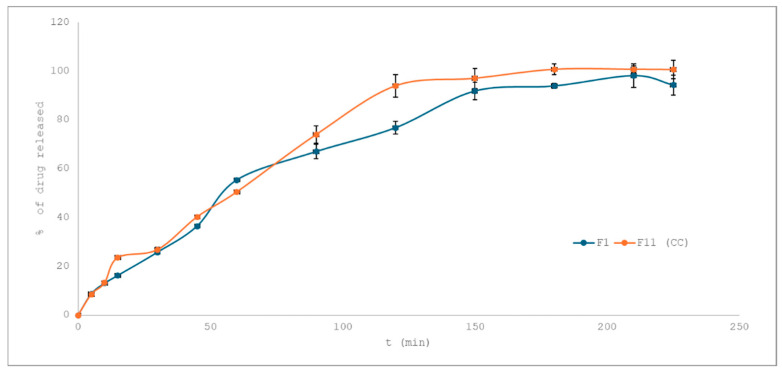
Drug release profiles from formulation F1 (without superdisintegrant) and F11 (with 1% of croscarmellose sodium).

As shown in Figure 6, both profiles exhibit gradual drug release beyond 120 min, F11 reached nearly complete drug release (100.78%), while F1 plateaued at 94.30% after 225 min. The calculated similarity factor (f_2_ = 58.91) indicates that the two profiles are similar (f_2_ > 50), though the presence of 1% croscarmellose sodium had a slight impact on enhancing release kineticsin the later stages of dissolution.

To further elucidate the release mechanism, dissolution data were fitted to commonly used mathematical models. Both F1 and F11 showed the best fit with the Korsmeyer–Peppas model (R^2^ = 0.97 for F1 and 0.98 for F11), suggesting a diffusion-controlled release, potentially accompanied by matrix erosion. The Higuchi model also yielded high determination coefficients (R^2^ ≈ 0.96), reinforcing the predominance of diffusion. The overall release mechanism did not change significantly with the addition of the superdisintegrant.

**Figure 7:**
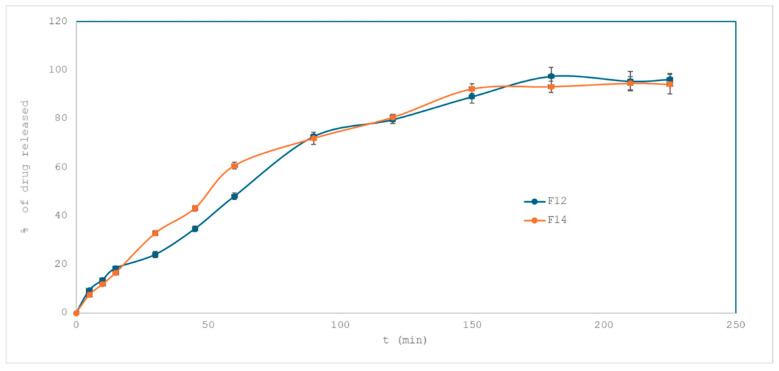
Drug release profiles from formulation F12 (with 3% croscarmellose sodium) and F14 (with 1% of croscarmellose sodium); both formulations with 5% of losartan potassium.

In the second comparison (Figure 7), F12 and F14—both with a reduced drug loading of 5% were evaluated. These formulations differed in the concentration of croscarmellose sodium: 3% in F12 and 1% in F14. As illustrated in Figure 6, F14 exhibited faster drug release in the first 60 min. However, after this point, both profiles converged, reaching similar levels of cumulative release by the end of the test (96.23% for F12 and 94.23% for F14). The calculated f_2_ value of 63.77 confirms high similarity, indicating that increasing the superdisintegrant concentration beyond 1% did not proportionally enhance the release rate, suggesting a plateau effect in its impact within this system.

In terms of release kinetics, both F12 and F14 were best described by the Korsmeyer–Peppas model (R^2^ = 0.97 for F12 and 0.99 for F14). The Higuchi model and zero-order model also showed high but slightly lower R^2^ values, supporting the dominance of diffusion as the primary mechanism. These results indicate that the mode of drug release remained consistent regardless of the superdisintegrant level. The plateau effect observed with croscarmellose sodium concentrations above 1% can be explained by the mechanism of superdisintegrant action in a hydrogel matrix. Croscarmellose sodium promotes water uptake by rapid swelling and capillary (wicking) action, which disrupts the polymer network and accelerates tablet disintegration. However, once a sufficient concentration is present to initiate efficient hydration and matrix rupture, the disintegration process becomes governed by the polymeric scaffold (HPMC) and drug solubility rather than by additional croscarmellose sodium. The plateau effect is consistent with previous reports on disintegrant optimization in hydrogel-based or 3D printed matrices, where an optimal range (commonly 1–2%) provides maximal benefit, while higher levels yield no further improvement or may even impair uniform printability.

Interestingly, despite comparable tablet mass and external surface area, the differences in dissolution behavior did not directly correlate with surface area, suggesting that internal structure and the role of the superdisintegrant in promoting hydration and matrix disintegration are more critical to drug release than external geometry alone.

Bilayer tablets of clopidogrel prepared using direct compression method formulated with croscarmellose sodium have shown good controlled release and follow first-order release and drug release by diffusion process based on Fick’s law of diffusion [41].

In conclusion, the addition of a superdisintegrant can slightly accelerate drug release, but at higher concentrations it does not proportionally enhance the release rate. The release mechanism remains diffusion-controlled and is best described by the Korsmeyer–Peppas model across all formulations. These findings underscore the importance of formulation optimization even in geometrically identical 3D-printed dosage forms.

#### 2.3.3. Effect of Drug Loading on Drug Release

**Figure 8:**
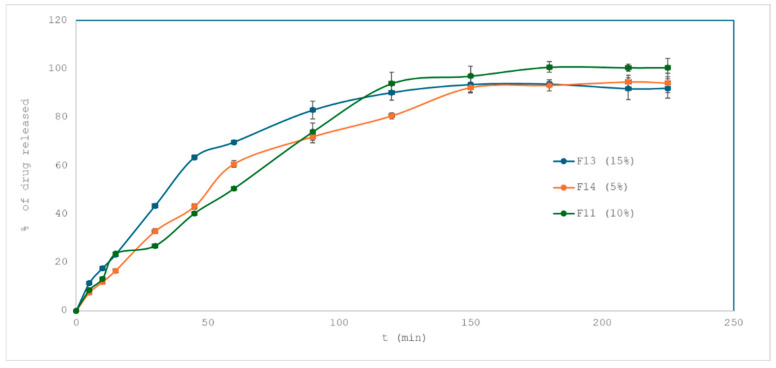
Drug release from formulations F13 (15% of drug loading), F14 (5%) and F11 (10%).

The influence of drug loading on the release profile of losartan potassium from 3D-printed tablets was evaluated by comparing formulations F13, F11, and F14, containing 15%, 10%, and 5% of the active substance, respectively. All three formulations were composed of the same excipients, including 1% croscarmellose sodium, and were printed using the same process parameters and tablet geometry. The dissolution profiles are shown in Figure 8.

Formulation F13 (15% drug loading) demonstrated the fastest initial release, with over 63% of the drug released within 45 min. In contrast, F14 (5%) reached only 43% at the same time point, while F11 (10%) released approximately 40%. Interestingly, F13 and F11 exhibited very similar behavior beyond 90 min, both reaching over 90% drug release by the end of the test. F14 reached 94.23% at 225 min, indicating slightly slower but complete release.

To quantify the similarity between profiles, the f_2_ similarity factor was calculated. The values were as follows:

- F13 vs. F11 (15% vs. 10%): f_2_ = 48.27 → profiles are not similar.
- F14 vs. F11 (5% vs. 10%): f_2_ = 59.33 → similar.
- F13 vs. F14 (15% vs. 5%): f_2_ = 53.53 → similar.

These results suggest that increasing drug loading to 15% significantly accelerates the early-stage release, producing a notably different dissolution profile from the 10% formulation. On the other hand, reducing the drug content from 10% to 5% slows the release moderately, but not enough to generate a dissimilar profile according to regulatory criteria.

Tablets with higher drug loading (15%) showed faster initial release, but the overall profile remained diffusion-controlled with complete release in about 2 h, indicating no uncontrolled dose dumping. While this behavior is acceptable for losartan, which has a broad therapeutic window, such accelerated early release could present a risk for drugs with a narrow therapeutic index and may require formulation adjustment.

Mathematical modeling of the dissolution data further supports these observations. All three formulations were best described by the Korsmeyer–Peppas model, with R^2^ values of 0.98 (F13), 0.99 (F11), and 0.99 (F14), indicating a consistent diffusion-controlled release mechanism. The Higuchi model also showed good fit (R^2^ > 0.96), confirming diffusion as the predominant mode of transport across all drug loadings.

Interestingly, despite the consistent release mechanism, the release kinetics were clearly affected by the drug content, particularly in the early stages of dissolution. This is likely due to increased osmotic pressure and matrix hydration gradients at higher drug loadings, which facilitate faster drug diffusion from the hydrogel network.

While the mechanism of release remains unchanged, the rate of release is sensitive to drug loading, with higher concentrations enhancing early drug liberation. This highlights the importance of balancing drug content and matrix composition during formulation design in order to achieve desired release profiles in personalized 3D-printed dosage forms.

### 2.4. Disintegration

Disintegration testing revealed notable differences among formulations depending on the presence of superdisintegrant, drug loading, and geometry. Formulations F11 (10% drug + 1% croscarmellose sodium) and F13 (15% drug + 1% croscarmellose sodium) exhibited the shortest disintegration times—25 and 40 min, respectively—suggesting that higher drug loading may weaken the hydrogel matrix formed by HPMC, thereby facilitating more rapid structural breakdown. In contrast, formulations F12 and F14, both containing 5% drug and either 3% or 1% croscarmellose sodium, disintegrated in 50 min, indicating that higher concentrations of superdisintegrant did not proportionally enhance disintegration at lower drug loading.

Formulations F1–F4, which lacked superdisintegrant, disintegrated significantly more slowly (55–60 min), regardless of geometry. These results indicate that in the absence of a functional disintegrant, shape alone does not substantially affect tablet disintegration when using HPMC-based matrices. Notably, all superdisintegrant-containing tablets were printed in the same geometry (rounded rectangle), thereby isolating formulation effects from geometric influences (Table 6).

The average disintegration time for formulations without superdisintegrant (F1–F4) was 56.25 ± 2.17 min, with minimal variation (CV% ~ 3.86%), confirming consistently slow disintegration.

In contrast, formulations with croscarmellose sodium (F11–F14) showed a lower average disintegration time of 41.25 ± 10.41 min, but with higher variability (CV% ~ 25.23%), likely influenced by drug loading differences and disintegrant concentration.

A parallel can be drawn between disintegration times and drug release behavior: the fastest-disintegrating formulation (F11) also exhibited the highest and most rapid drug release, while formulations with slower disintegration (F12, F14) demonstrated more gradual dissolution. However, despite F13 disintegrating slower than F11, its higher drug loading compensated by accelerating release kinetics, supporting the hypothesis that both matrix disruption and osmotic gradients contribute to the drug liberation process. These findings confirm that formulation composition—particularly the interplay between polymer content, drug loading, and disintegrant level—plays a more significant role than geometry in determining disintegration performance in SSE-printed tablets.

### 2.5. FT-IR Analysis

FT-IR spectroscopy was utilized to investigate the compatibility between the active pharmaceutical ingredient (API) and excipients used in tablet formulation, given that intermolecular interactions can significantly impact the stability of formulations and the physical state of the API. Using FT-IR, the presence of intermolecular interactions can be identified based on shifts in the absorption frequencies of functional groups within the molecules under investigation [42].

**Figure 9:**
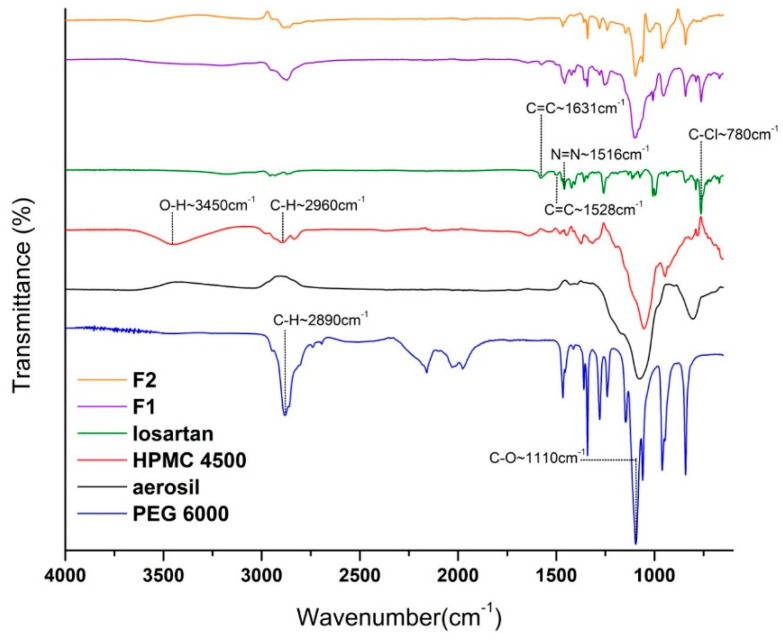
FT-IR spectra of active substance (losartan potassium), excipients (HPMC 4500, PEG 6000, and aerosil) and the formulated tablets (F1 and F2 formulations).

The FT-IR spectra of active substance and excipients (losartan potassium, HPMC 4500, PEG 6000, and aerosil (SiO_2_)) and the formulated tablets are shown in following Figure 9:

In the FT-IR spectrum of pure losartan potassium, characteristic peaks are observed at 780 cm^−1^ (C–Cl stretching), 1516 cm^−1^ (N=N stretching vibrations), and 1528 and 1631 cm^−1^ (C=C stretching), consistent with findings by Franca et al. (2006) [43]. A broad absorption band is present at 3198 cm^−1^, with bands at 1046 and 1157 cm^−1^ from the tetrazole ring and a sharp band at 1528 cm^−1^ attributed to the imidazole ring. Significant vibrations noted in the PEG 6000 spectrum include C–H stretching at 2890 cm^−1^ and C–O stretching at 1110 cm^−1^; similar positions are observed in the FT-IR spectra of the manufactured tablets. The spectrum of HPMC shows absorption bands at 3450 cm^−1^ (O–H stretching) and 2960 cm^−1^ (C–H stretching), consistent with the substituent analysis and rheological properties described by Akinosho et al. (2013) [44]. The intense absorption band at 1060 cm^−1^ corresponds to out-of-phase vibrations associated with alkyl-substituted cyclic rings containing ether bonds, as also observed by Bashir et al. (2020) [45] in hydroxypropyl methylcellulose (HPMC)-based systems.

All characteristic peaks of losartan potassium were observed at identical frequencies in both the pure losartan potassium FT-IR spectrum and the spectra of formulated tablets. These findings suggest that no strong chemical interactions (e.g., hydrogen bonding, salt formation, or esterification) occurred between the drug and excipients during formulation and printing. The integrity of the losartan potassium structure was maintained, indicating that the SSE printing process and formulation environment did not induce chemical instability. Future work will include solid-state characterization to confirm possible polymorphic transitions or amorphization induced by SSE.

### 2.6. Process Development and Possible Scale-Up

Quality by Design (QbD) framework was adopted to identify and control the Critical Material Attributes (CMAs) and Critical Process Parameters (CPPs) affecting the Critical Quality Attributes (CQAs) of the final 3D-printed product. This risk-based approach ensures printabiliy, reproducibility and robustnes of semi-solid formulations and SSE printing process. Detailed QTPP, CMAs, CPPs, and CQAs analysis is presented in Table 7, Table 8, Table 9 and Table 10, indicating a scalable process, possible for future translation to larger-scale manufacturing.

The selected dosage form (solid tablet) was designed for peroral administration, aligning with conventional patient preferences and regulatory familiarity. The use of SSE 3D printing enabled the fabrication of various tablet geometries, including rounded rectangles, triangles, circles, and doughnut-shaped forms. These shapes were chosen to explore the potential for geometry driven modulation of drug release, as well as to enhance visual appeal and support personalization strategies.

Dimensional constraints were maintained within acceptable limits for oral administration, with tablet sizes ranging from 15 mm to 22 mm in length and up to 5 mm in thickness. This ensured both dose capacity and swallowability were considered in the design phase. The doughnut shape, in particular, introduces a central void that increases surface area and may accelerate dissolution, highlighting the potential of shape-engineering to influence pharmacokinetics.

The targeted dissolution profile was defined across three time points: ≥20% at 1 h (L1), ≥50% at 2 h (L2), and >80% at 3 h (L3), to reflect a modified or extended-release intent, suitable for APIs requiring prolonged therapeutic coverage.

Mechanical robustness was also incorporated into the QTPP, with the requirement that printed tablets exhibit no cracking or deformation following the drying process.

The solubility of the active ingredient in the hydroalcoholic vehicle was recognized as a key CMA due to its direct impact on dissolution behavior. To ensure consistent solubilization and prevent precipitation during processing, a fixed ethanol–water ratio was selected, along with temperature-controlled mixing. This controlled environment ensured reproducible active ingredient distribution within the semi-solid matrix and contributed to the robustness of the printing process.

Polyethylene glycol (at 15% *w*/*w*) was identified as a multifunctional excipient influencing plasticity, flowability, and ultimately, printability. Its concentration was optimized through preliminary screening studies to achieve a balance between extrusion ease and shape retention post-printing.

Hydroxypropyl methylcellulose (HPMC) with a viscosity grade of 4500 cps was used as a matrix-forming polymer, critical for gel formation and print fidelity. The viscosity grade was chosen and optimized through preliminary screening studies to ensure adequate structural integrity.

The inclusion of silicon dioxide (SiO_2_) at 1% *w*/*w* served to enhance thixotropic behavior and prevent phase separation within the semi-solid formulation. Its concentration was fixed and was not varied during the development and production phases.

The optimization and control of Critical Process Parameters (CPPs) were essential to ensure consistent product quality during the semi-solid extrusion (SSE) 3D printing process. Each parameter was identified based on its potential influence on Critical Quality Attributes (CQAs).

Mixing temperature was controlled at approximately 35 °C (±2 °C) to facilitate the complete solubilization of the active ingredient and sufficient to reduce viscosity and promote homogeneous blending without compromising the thermal stability of the formulation components.

The stirring speed and duration were recognized as key CPPs, affecting both ingredient dispersion and the incorporation of air bubbles since inadequate mixing could result in drug entrapped air, which can compromise both content uniformity and structural integrity of the printed dosage form.

Following mixing, a gelation or resting period of 10 min at room temperature (22 °C) was introduced to allow for air bubble release and to promote gel structure development. This step improved the extrusion behavior.

The method of syringe filling was standardized and performed manually using a consistent protocol to minimize air entrapment and ensure uniform loading, avoiding print defects associated with air pockets.

Extrusion speed and pressure were pre-defined in the printer’s G-code and verified before each printing run. These parameters directly influenced the quality of deposited layers, dimensional accuracy, and print continuity.

Nozzle diameter was fixed (0.84 mm) across all formulations to maintain consistent resolution and flow rate during printing. Variability in nozzle size could significantly impact the thickness of printed layers and the printing precision.

Finally, drying conditions (40 °C for 4 h) were standardized to ensure adequate removal of residual moisture and structural integrity of the printed tablets. This post-processing step is particularly critical in SSE-printed products, where mechanical strength is reliant on both proper solvent removal and matrix consolidation.

The identification and control of Critical Quality Attributes (CQAs) ensure that the final 3D-printed dosage forms meet predefined product characteristics and quality standards.

Dosage unit mass was maintained within the specified range of 500 ± 5 mg, as verified by individual weighing of 12 units per batch. This attribute is essential for dose accuracy, particularly in individualized or small-batch manufacturing processes such as semi-solid extrusion (SSE) 3D printing.

Drug content across printed tablets was within 90–110% of the label claim, as quantified by UV/Vis spectrophotometry.

Dimensional accuracy, measured using a digital caliper, showed a coefficient of variation (CV) of less than 3% for both height and diameter across all tablet geometries, reflecting well-controlled CPPs.

Disintegration time was below the specified limit of 60 min, assessed per USP <701>. The achieved times were acceptable for the intended application.

Dissolution testing using USP Apparatus I (basket method) demonstrated compliance with the pre-established release thresholds. Results indicate that the selected formulation and geometric configurations support controlled drug release.

Mechanical integrity was confirmed via visual inspection, with no evidence of cracking, delamination, or shape collapse. The robustness of the tablets can be attributed to the inclusion of suitable excipients and optimized drying conditions.

Finally, surface quality was visually inspected and found to be smooth, with minimal layering defects. This outcome reflects the high print fidelity achieved under controlled extrusion parameters.

## 3. Materials and Methods

### 3.1. Materials

Losartan potassium (CAS No. 124750-99-8, ≥99% purity, Ph. Eur. grade), hydroxypropyl methylcellulose (HPMC 4500, CAS No. 9004-65-3, ≥99% purity, Ph. Eur. grade), and polyethylene glycol (PEG 6000, CAS No. 25322-68-3, ≥99% purity, Ph. Eur. grade) were obtained from Galenika A.D. (Belgrade, Serbia). Colloidal silicon dioxide (SiO_2_, CAS No. 7631-86-9, ≥99% purity, Ph. Eur. grade) and croscarmellose sodium (CAS No. 74811-65-7, ≥99% purity, Ph. Eur. grade) were provided by Merck KGaA (Darmstadt, Germany). HPMC (Benecel™) K35M (Ph. Eur. grade) was procured from Ashland Industries Europe GmbH (Schaffhausen, Switzerland), while HPMC 615 (Pharmacoat^®^ 615, Ph. Eur. grade) was supplied by Pharma Excipients (Zug, Switzerland).

#### 3.1.1. Preparation and Evaluation of Polymer–Solvent Mixtures for Printability Assessment

A series of polymer–solvent mixtures were prepared to evaluate their printability for semi-solid extrusion (SSE). Each polymer was gradually added in small portions into the solvent (either purified water or an ethanol–water mixture, considering the active substance solubility [46] to minimize lump formation and aim for homogenous consistency.

In selected formulations, croscarmellose sodium was added as a potential superdisintegrant either simultaneously with or after polymer addition, as indicated in Table 11, in order to analyze its impact on solution viscosity.

#### 3.1.2. Design of Printable Dosage Forms

Three-dimensional (3D) tablet designs were created using Tinkercad^®^ (Autodesk, Inc., San Francisco, CA, USA, https://www.tinkercad.com/), a web-based computer-aided design (CAD) tool compatible with semi-solid extrusion (SSE) printing. To investigate the effect of geometry on printability and potential drug release characteristics, four distinct shapes were developed: rounded rectangle, triangle, doughnut, and circle.

The dimensions, estimated volumes, and surface areas of each design are summarized in Table 12. Shapes varied in surface-area-to-volume ratios, which may influence both the mechanical stability during and after printing, as well as the rate of drug release from the matrix. All designs were printed in three layers to simulate typical unit doses. The rounded rectangle (20 × 12 × 4 mm) and round tablet (15 × 15 × 5 mm) were chosen as standard oral dosage geometries, while the triangle (22 × 15 × 5 mm) and doughnut (outer diameter 20 mm, inner diameter 10 mm, height 5 mm) served to evaluate non-conventional structures.

**Figure 10:**
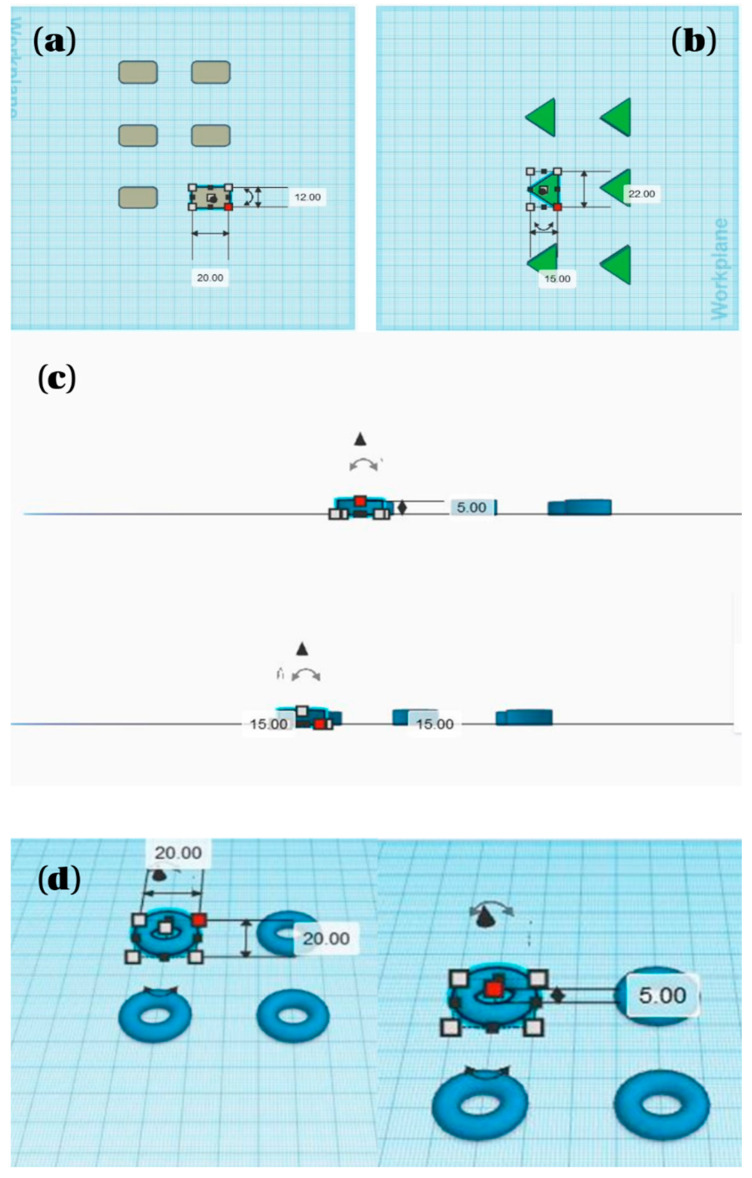
3D Designs of tablet shapes prepared for SSE printing (representative screenshots of designed tablet geometries created in Tinkercad^®^): (**a**) rounded rectangle (20 × 12 × 4 mm), (**b**) triangle (22 × 15 × 5 mm), (**c**) round tablet (15 × 15 × 5 mm), (**d**) doughnut (outer diameter: 20 mm; inner diameter: 10 mm; height: 5 mm).

Visual representations of the 3D designs are shown in Figure 10, illustrating their geometric profiles and dimensional scaling. These geometries were selected not only for their theoretical relevance to drug release modulation, but also to test the formulation’s ability to support various shape changes during SSE printing.

Each design was exported in STL format and sliced for layer-by-layer extrusion printing. The shapes were selected to evaluate the effect of geometry on printability, mechanical stability, and drug release potential.

#### 3.1.3. Preparation of Drug-Loaded Formulations for SSE Printing

The composition of losartan potassium-containing formulations designed for SSE 3D printing is shown in Table 13. HPMC 4500 was selected as the primary matrix-forming polymer based on its demonstrated suitability for semi-solid extrusion (see Section 3.1.1). To enhance formulation performance, two additional excipients were incorporated. Polyethylene glycol 6000 (PEG 6000) was included at 15% (*w*/*w*) as a multifunctional agent. As a co-solvent, PEG 6000 improves the solubility and dispersion of losartan potassium in the hydroalcoholic vehicle. Simultaneously, it acts as a plasticizer, improving the flexibility and cohesion of the printed layers and minimizing cracking or structural failure during drying. Colloidal silicon dioxide (SiO_2_) was added at 1% (*w*/*w*) to increase the viscosity and thixotropy of the formulation. Its inclusion stabilizes the semi-solid matrix, prevents phase separation during rest, and contributes to the dimensional stability of printed constructs.

**Figure 11:**
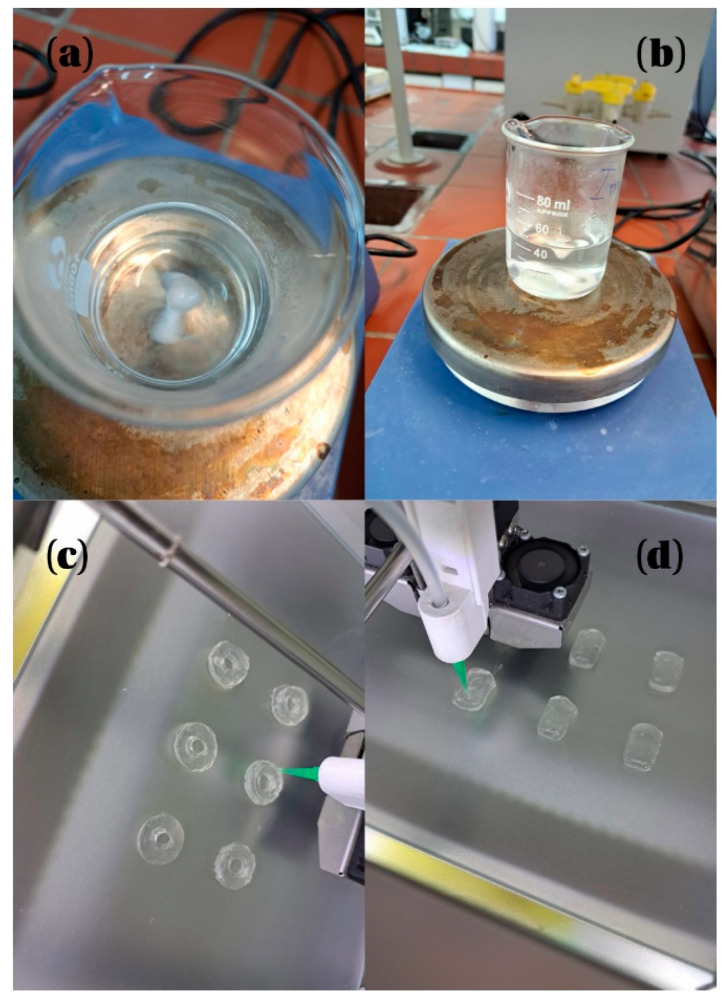
SSE process: (**a**,**b**) mixing of the tablet formulation, (**c**) 3D printing of tablets with a doughnut shape, (**d**) 3D printing of tablets with a rounded rectangular shape.

The required amount of solvent (ethanol-water mixture) was measured into a beaker and placed on a magnetic stirrer (IKA-Werke GmbH & Co. KG, Staufen, Germany). At ~35 °C and a moderate speed losartan potassium was dissolved first (mean time: 1.0 ± 0.3 min), followed by PEG 6000 and SiO_2_ (3.5 ± 0.2 min). The stirring speed was then increased, and polymer was added in small portions to prevent bubbling in the viscous solution (8.8 ± 0.5 min) (Figure 11). The beaker was covered with a watch glass to reduce losses due to evaporation. The solution was left at 22.3 ± 0.5 °C for ten minutes to eliminate air bubbles and allow gel formation.

This process step showed high reproducibility across three independent batches, as evidenced by the low standard deviations, suggesting a well-controlled and repeatable mixing process, which is critical for achieving consistent rheological properties in SSE-based 3D printing.

#### 3.1.4. Three-dimensional Printing Procedure Using Semi-Solid Extrusion (SSE)

The drug-loaded semi-solid formulation was manually loaded into a disposable 60 mL plastic syringe (Thermo Fisher Scientific Inc., Vantaa, Finland), which served as the extrusion reservoir (5.4 ± 0.4 min). To eliminate entrapped air and ensure proper flow continuity, the syringe plunger was gently pressed until a small amount of formulation emerged from the nozzle tip. The prepared syringe was then mounted onto the Ultimaker 2+ 3D printer (Ultimaker B.V., Geldermalsen, The Netherlands), modified for semi-solid extrusion and equipped with a stainless steel 0.8 mm nozzle (6.6 ± 0.3 min). Printing was carried out at a nozzle temperature of 80 °C and a build plate temperature of 25 °C, with an extrusion flow rate of 220%. These settings were selected to balance formulation viscosity, solvent evaporation, and structural fidelity and the results of preliminary experiments

Nozzle temperature is below the melting temperature of the active substance (183.5–184.5 °C [47]). The slicing and G-code generation were performed using Ultimaker Cura software (version 4.13.1). Detailed printing parameters are summarized in Table 14.

Each formulation was printed layer by layer to achieve the target 3D geometry as defined in the STL models (see Section 3.1.1). Printing time for all formulations was in 12–15 min range (depending on formulation, the printing time is fixed and printer controlled). Following printing, the constructs were left on the build plate overnight at ambient conditions (~22 °C) to allow for passive solvent evaporation and complete structural solidification.

The printing process is performed under controlled conditions (printing parameters, flow rate, nozzle temperature, build plate temperature, ambient temperature) and ensures a high level of reproducibility and operational control during semi-solid extrusion.

#### 3.1.5. Characterization of Printed Tablets

##### Uniformity of Mass and Dimensions

The mass uniformity of printed tablets was evaluated by individually weighing 12 units per formulation using an analytical balance (Kern & Sohn GmbH, Balingen-Frommern, Germany). The mean values and standard deviations were calculated.

Dimensional uniformity was assessed by measuring the diameter and thickness of 10 tablets per formulation using a digital caliper (Vogel, Kevelaer, Germany). These measurements were used to evaluate the reproducibility and accuracy of the 3D printing process in maintaining the intended geometric specifications.

##### Drug Release

In vitro dissolution studies were carried out using an ERWEKA DT 600 dissolution tester (ERWEKA GmbH, Langen, Germany) equipped with rotating baskets (USP Apparatus 1). Each vessel contained 500 mL of purified water, maintained at 37 ± 0.5 °C. The printed tablets (n = 6) were placed in baskets and rotated at 100 rpm. Aliquots of 4 mL were withdrawn at predefined time intervals (5, 15, 30, 45, 60, 90, 120, 180, and 210 min), filtered, and analyzed without replacing the medium.

500 mL of purified water was selected as the dissolution medium for losartan potassium based on its high aqueous solubility, neutral pKa, and preliminary solubility profiling, which confirmed that losartan potassium dissolves readily in water at 37 ± 0.5 °C.

USP Apparats 1 was used due to potential drug product floating characteristics. The 100 rpm paddle speed was selected based on the physical characteristics of the 3D-printed semi-solid extrusion (SSE) dosage forms, which differ from conventional compressed tablets in terms of density, porosity, and surface area. Due to their lighter weight lower paddle speeds (e.g., 50 rpm, USP) resulted in floating or incomplete agitation during preliminary trials.

Predifined time intervals were set to better monitor the drug release kinetics and obtain a complete dissolution profile.

Drug concentration was determined by measuring the absorbance at 250 nm [47] using a UV–Vis spectrophotometer (Evolution 300, Thermo Fisher Scientific, Cambridge, UK), using calculation via standard solution.

The resulting dissolution profiles were compared by calculating the similarity factor *f*_2_, a logarithmic reciprocal square root transformation of the sum of squared errors between the test and reference products over all time points:

$$f_{2}=\;50\;\cdot log\;\left\{\;{\left.\left[1\;+\;\frac{1}{n}\sum {\left(R_{t}-\;T_{t}\right)}^{2}\right.\right]}^{\left(-0.5\right)}\cdot 100\;\right\}$$

where *Rt* and *Tt* represent the percentage of drug dissolved at each time point for the reference and test products, respectively, and n is the number of time points. Values of *f*_2_ between 50 and 100 suggest similarity between the compared profiles.

Furthermore, to interpret the drug release mechanism, the dissolution profiles were fitted to several mathematical models: 

Zero-order model:

$$Q_{t}=\;Q_{0}+\;k_{0}\cdot t$$

where

- *Qₜ*: amount of drug released at time t
- *Q*_0_: initial amount of drug (usually zero)
- *k*_0_: zero-order rate constant

First-order model:

$$\log Q_{t}=\log Q_{0}-\frac{\left(k_{1}*\;t\right)}{2.303}$$

where

- *Qₜ*: amount of drug remaining at time t
- *Q*_0_: initial amount of drug
- *k*_1_: first-order rate constant

Higuchi model:

$$Q_{t}=\;k_{H}\cdot \sqrt{\left(t\right)}$$

where

- *Qₜ*: amount of drug released at time t
- *k_H_*: Higuchi dissolution constant
- *t*: time

Korsmeyer–Peppas model:

$$\frac{Q_{t}}{Q_{\infty }}=\;k_{K}\cdot t^{n}$$

where

- *Qₜ*: amount of drug released at time t
- *Q∞*: total amount of drug released at infinite time
- *k_K_*: kinetic constant
- *n*: release exponent (indicates mechanism of drug release)

Hixson–Crowell model:

$$Q_{0}^{\frac{1}{3}}-\;Q_{t}^{\frac{1}{3}}=\;k_{HC}\cdot t$$

where

- *Q*_0_: initial amount of drug
- *Qₜ*: amount of drug remaining at time t
- *k_HC_*: Hixson–Crowell rate constant
- *t*: time

Fitting these models to the experimental data enables deeper understanding of the mechanisms governing drug release from different tablet geometries.

##### Disintegration Testing

Disintegration tests were performed using an Erweka ZT 52 disintegration tester (ERWEKA GmbH) in 800 mL of purified water, maintained at 37 ± 0.5 °C, over a total duration of 2 h. Tablets were visually monitored for complete disintegration, defined as the absence of any remaining intact core structure on the apparatus screen.

##### Fourier-Transform Infrared Spectroscopy (FT-IR)

FT-IR spectroscopy was employed to identify potential interactions between losartan potassium and excipients within the printed matrix. Spectra were recorded using a Nicolet iS10 FT-IR spectrometer (Thermo Scientific, Waltham, MA, USA), equipped with an attenuated total reflectance (ATR) accessory (Smart iTR) and a zinc selenide (ZnSe) crystal.

Samples analyzed included powdered 3D-printed tablets, pure losartan potassium, and individual excipients. Spectra were collected over a wavenumber range of 4000–650 cm^−1^ at a resolution of 2 cm^−1^. The presence or absence of characteristic peaks, as well as any peak shifts, were used to evaluate possible intermolecular interactions or structural changes upon formulation.

## 4. Conclusions

This study demonstrated that semisolid extrusion (SSE) 3D printing can be effectively applied to fabricate personalized oral dosage forms containing losartan potassium by systematically evaluating formulation composition, printing parameters, and geometry. The optimal polymer–solvent system (HPMC 4500 with PEG 6000 and ethanol–water mixture) enabled reliable printability, shape retention, and rapid disintegration, with the addition of 1% croscarmellose sodium producing the most significant enhancement in drug release without further improvement at higher concentrations. The influence of tablet geometry was generally minimal; however, the doughnut shape showed a lower f_2_ similarity value (~52.5) compared to other designs, likely due to differences in internal void structure and surface area-to-volume ratio. Increased drug loading (15%) accelerated initial dissolution, which may require formulation balancing to prevent potential dose dumping in immediate-release profiles.

A comprehensive Quality Target Product Profile (QTPP), which considered dose flexibility and precision, robust mechanical properties of the dosage forms, and controlled drug release profiles is in line with the aims of this study and therapeutic goals. Critical Quality Attributes (CQAs) such as disintegration time, mechanical integrity, and drug release kinetics were systematically monitored and optimized to ensure product efficacy and support patient compliance.

Key Critical Material Attributes (CMAs), including the type and concentration of matrix formers, superdisintegrants, and drug loading, were carefully selected based on their physicochemical compatibility and their influence on the hydrogel matrix formation and tablet dissolution behavior. These CMAs played a significant role in drug release rate and tablet integrity.

Critical Process Parameters (CPPs), such as extrusion speed and nozzle diameter, were optimized through Design of Experiments (DoE) to achieve consistent filament extrusion and uniform layer formation. This ensured reproducibility across batches, which is crucial for regulatory compliance and clinical performance.

By systematically integrating QTPP, CQAs, CMAs, and CPPs within a Quality by Design (QbD) framework, the study gained comprehensive qualitative and quantitative understanding of the critical relationships between formulation variables and processing parameters. This strategy facilitated precise customization of tablet geometry, composition, and printing conditions to achieve targeted drug release profiles, highlighting the versatility and reliability of 3D printing technology for personalized delivery of losartan potassium.

The combination of f_2_ analysis and statistical testing confirmed that the observed differences in dissolution and disintegration were formulation- and geometry-dependent. FT-IR analysis indicated no significant drug–excipient interactions, supporting the physical stability of the formulations. These findings confirm that SSE 3D printing can produce individualized dosage forms meeting pharmacopeial requirements, with flexibility in dose, geometry, and excipient composition. From a regulatory perspective, the demonstrated reproducibility, process control, and material characterization underline the potential of SSE to be integrated into GMP-compliant manufacturing workflows, supporting its application in personalized medicine.

## Figures and Tables

**Table 1:** Characterization of Polymer–Solvent Mixtures (With/Without Superdisintegrant).

Polymer/Croscarmellose Sodium/Solvent	Concentration (% *w*/*w*)	Observations
Benecel K35M + Croscarmellose sodium/Water	1% + 1%	Exhibited excessively low viscosity.
Benecel K35M + Croscarmellose sodium/Water	2% + 2%	Optimal viscosity; high presence of bubbles (disappear with settling).
Benecel K35M + Croscarmellose sodium/Water	2% + 4%	Fewer bubbles present; increased viscosity (printed with flow rate 280%); shape spreading during print (poor plastic properties).
HPMC 615 + Croscarmellose sodium/Water	10% + 1%	High bubble presence; optimal viscosity; shape spreading and poor adhesion between layers.
HPMC 615/Water	12%	High viscosity and bubble presence; unsuitable for printing.
HPMC 615/Ethanol–water (50:50 *v*/*v*)	12%	Inhomogeneous; mixing difficulties.
HPMC 615 + Croscarmellose sodium/Ethanol–water (50:50 *v*/*v*)	10% + 1%	Inhomogeneous; mixing difficulties.
HPMC 4500 + Croscarmellose sodium/Ethanol–water (9:1 *v*/*v*)	5% + 1%	Slight mixing difficulties; suitable viscosity and flow; successful harder form.

**Table 2:** Used matrix-former in case of all batches of 3D printed losartan potassium tablets.

Excipient	Role	F1	F2	F3	F4	F11	F12	F13	F14
HPMC 4500	binder	x	x	x	x	x	x	x	x
PEG 6000	binder	x	x	x	x	x	x	x	x
SiO_2_	plasticizer	x	x	x	x	x	x	x	x
Croscarmellose sodium	disintegrant	-	-	-	-	x	x	x	x
Ethanol–water (9:1 *v*/*v*)	solvent	x	x	x	x	x	x	x	x

**Table 3:** Obtained dimensional values (mm) of printed tablets (n = 12), rectangular shape, F1.

Tablet No	Length (mm)	Width (mm)	Height (mm)
1	18.50	10.00	4.00
2	19.00	10.50	3.50
3	19.50	11.00	4.00
4	19.00	9.50	4.00
5	20.00	11.50	4.00
6	19.50	12.00	4.00
7	18.50	11.50	3.50
8	19.00	11.00	4.00
9	20.00	11.50	4.00
10	19.00	12.00	4.00
11	18.50	11.00	3.50
12	19.50	11.50	4.00
Average	19.17	11.08	3.87
SD	0.54	0.76	0.22

**Table 4:** Average Mass and Standard Deviation of Printed Tablets.

Formulation	Average Mass (g)	SD (g)	CV (%)
F1	0.425	0.026	6.12
F2	0.398	0.047	11.81
F3	0.489	0.063	12.89
F4	0.513	0.051	9.94
F11	0.573	0.060	10.47
F12	0.623	0.107	17.17
F13	0.821	0.116	14.13
F14	0.709	0.070	9.87

**Table 5:** R^2^ Values for Mathematical Models Applied to Drug Release Profiles.

Model	Rounded Rectangle	Triangle	Doughnut	Round Tablet
Zero-order	0.9165	0.9437	0.8632	0.9517
First-order	0.7886	0.8191	0.7335	0.8313
Higuchi	0.9649	0.9563	0.9641	0.9602
Korsmeyer–Peppas	0.9747	0.9809	0.9641	0.9863
Hixson–Crowell	0.8309	0.8623	0.7742	0.8733

**Table 6:** Disintegration Times for 3D-Printed Tablets.

Formulation	Geometry	Drug Loading (%)	Superdisintegrant	Conc. (%)	Disintegration Time (min)
F1	Rounded Rectangle	5	None	0	55
F2	Triangle	5	None	0	55
F3	Doughnut	5	None	0	55
F4	Round	5	None	0	60
F11	Rounded Rectangle	10	Croscarmellose Sodium	1	25
F12	Rounded Rectangle	5	Croscarmellose Sodium	3	50
F13	Rounded Rectangle	15	Croscarmellose Sodium	1	40
F14	Rounded Rectangle	5	Croscarmellose Sodium	1	50

**Table 7:** Quality Target Product Profile (QTPP).

Quality Target Product Profile (QTPP)	Shape
Dosage Form	3D SSE printed tablet
Route of Administration	oral
Appearance	Solid, tablet with smooth layers rounded rectangle, triangle, doughnut or circle shape
Dimensions	Rounded rectangle 20 × 12 × 4 mm Triangle 22 × 15 × 5 mm Doughnut 20 × 20 × 5 mm (outer ⌀) 10 mm (inner ⌀) Circle 15 × 15 × 5 mm
Dissolution profile	L1: 20% in 1 h L2: 50% in 2 h L3: >80% in 3 h
Mechanical Strength	No cracking or shape collapse post-printing/drying

**Table 8:** Critical Material Attributes (CMAs).

Critical Material Attributes (CMAs)	Impact on CQAs	Control Strategy
API solubility in hydroalcoholic vehicle	Affects drug content uniformity and release rate	Fixed solvent ratio (ethanol:water), temperature-controlled dissolution
PEG 6000 content (15% *w*/*w*)	Affects plasticity, flowability, and printability	Optimized based on prior screening
HPMC viscosity (4500 cps)	Impacts gel formation, print fidelity, and layer adhesion	Pre-characterized polymer
SiO_2_ (1% *w*/*w*)	Improves thixotropy and prevents phase separation	Fixed concentration

**Table 9:** Critical Process Parameters (CPPs).

Critical Process Parameters (CPPs)	Impact on CQAs	Control Strategy
Mixing temperature (~35 °C)	Solubilization of API, blend uniformity	Maintained ± 2 °C using hotplate/stirrer
Stirring duration and speed	API dispersion, bubble formation	Time and speed controlled mixing
Gelation/resting time (10 min at 22 °C)	Air removal, shape formation	Constant across batches
Syringe filling method	Uniform loading, air bubble prevention	Manual, standardised
Extrusion speed/pressure	Layer quality, dimensional accuracy	Pre-set in G-code, verified before runs
Nozzle diameter (e.g., 0.84 mm)	Affects resolution and flow	Constant
Drying conditions (e.g., 40 °C/4 h)	Mechanical integrity, residual moisture	Fixed drying protocol validated for shape retention

**Table 10:** Critical Quality Attributes (CQAs).

Critical Quality Attributes (CQAs)	Specification/Target	Test Method
Dosage unit mass	500 ± 5 mg	Mass variation by individually weighing 12 units per formulation
Drug content	90–110% of label claim	UV/Vis spectrophotometry
Dimensional accuracy	CV < 3% for height and diameter	Digital caliper measurement
Disintegration time	<60 min	USP <701>
Dissolution profile	L1: 20% in 1 h L2: 50% in 2 h L3: >80% in 3 h	USP Apparatus I (basket)
Mechanical integrity	No cracking or delamination	Visual assessment
Surface quality	Smooth, no visible layering defects	Visual inspection

**Table 11:** Composition of formulations with different polymer–solvent mixtures evaluated for printability (losartan potassium 10% *w*/*w* and PED 6000 15% unchanged, solvent ad 20 g).

Mixture Code	Polymer/Superdisintegrant/Solvent	Concentration (%*w*/*w*)	Quantity (g)
M1	Benecel K35M + Croscarmellose sodium/Water	1% + 1%	0.2 + 0.2
M2	Benecel K35M + Croscarmellose sodium/Water	2% + 2%	0.4 + 0.4
M3	Benecel K35M + Croscarmellose sodium/Water	2% + 4%	0.4 + 0.8
M4	HPMC 615 + Croscarmellose sodium/Water	10% + 1%	2.0 + 0.2
M5	HPMC 615/Water	12%	2.4
M6	HPMC 615/Ethanol–water (50:50 *v*/*v*)	12%	2.4
M7	HPMC 615 + Croscarmellose sodium/Ethanol–water (50:50 *v*/*v*)	10% + 1%	2.0 + 0.2
M8	HPMC 4500 + Croscarmellose sodium/Ethanol–water (9:1 *v*/*v*)	5% + 1%	1.0 + 0.2

**Table 12:** Geometrical Properties and Surface-Area-to-Volume Ratios of Printed Tablet Designs.

Shape	Dimensions	Volume (mm^3^)	Surface Area (mm^2^)	SA:V Ratio (mm^−1^)
Rounded Rectangle	20 × 12 × 4 mm	946.3	736.0	0.778
Triangle	22 × 15 × 5 mm	825.0	648.1	0.786
Doughnut	20 × 20 × 5 mm (outer ⌀) 10 mm (inner ⌀)	1178.1	942.5	0.8
Circle	15 × 15 × 5 mm	883.6	589.0	0.667

**Table 13:** Composition of Formulations (Quantities in % *w*/*w*).

Component	F1	F2	F3	F4	F11	F12	F13	F14
Losartan potassium	10	10	10	10	10	5	15	5
HPMC 4500	5	5	5	5	5	5	5	5
PEG 6000	15	15	15	15	15	15	15	15
SiO_2_	1	1	1	1	1	1	1	1
Croscarmellose sodium	-	-	-	-	1	3	1	1
Ethanol–water (9:1 *v*/*v*)	69	69	69	69	68	71	63	73
Tablet shape	RR	T	D	C	RR	RR	RR	RR

RR—Rounded rectangle, T—triangle, D—Doughnut, C—Circle.

**Table 14:** Printing Parameters Used in Ultimaker Cura 4.13.1.

Parameter	Value
Layer Height	1.2 mm
Top/Bottom Thickness	1.2 mm
Top/Bottom Layers	1/1
Infill Density	100%
Infill Pattern	Lines
Infill Layer Thickness	1.2 mm
Print Speed	15.0 mm/s
Travel Speed	80.0 mm/s
Initial Layer Speed	11.0 mm/s
Fan Speed	100%
Build Plate Adhesion	None
Adaptive Layers	No

## Data Availability

The original contributions presented in this study are included in the article. Further inquiries can be directed to the corresponding author.

## Abbreviations

The following abbreviations are used in this manuscript:

3D	Three-dimensional
SSE	Semi-solid extrusion
HPMC	Hydroxypropyl methylcellulose
FDM	Fused deposition modeling
PEG	Polyethylene glycol
CAD	Computer-aided design
FT-IR	Fourier-Transform Infrared
API	Active Pharmaceutical Ingredient
